# A Method for Comparing the Impact on Carcinogenicity of Tobacco Products: A Case Study on Heated Tobacco Versus Cigarettes

**DOI:** 10.1111/risa.13482

**Published:** 2020-05-01

**Authors:** Wout Slob, Lya G. Soeteman‐Hernández, Wieneke Bil, Yvonne C.M. Staal, W. Edryd Stephens, Reinskje Talhout

**Affiliations:** ^1^ National Institute for Public Health and the Environment (RIVM) Bilthoven The Netherlands; ^2^ School of Earth & Environmental Sciences University of St. Andrews Scotland UK

**Keywords:** Carcinogenicity, cumulative exposure, heated tobacco, relative potency, tobacco products

## Abstract

Comparing the harmful health effects related to two different tobacco products by applying common risk assessment methods to each individual compound is problematic. We developed a method that circumvents some of these problems by focusing on the change in cumulative exposure (CCE) of the compounds emitted by the two products considered. The method consists of six steps. The first three steps encompass dose‐response analysis of cancer data, resulting in relative potency factors with confidence intervals. The fourth step evaluates emission data, resulting in confidence intervals for the expected emission of each compound. The fifth step calculates the change in CCE, probabilistically, resulting in an uncertainty range for the CCE. The sixth step estimates the associated health impact by combining the CCE with relevant dose‐response information. As an illustrative case study, we applied the method to eight carcinogens occurring both in the emissions of heated tobacco products (HTPs), a novel class of tobacco products, and tobacco smoke. The CCE was estimated to be 10‐ to 25‐fold lower when using HTPs instead of cigarettes. Such a change indicates a substantially smaller reduction in expected life span, based on available dose‐response information in smokers. However, this is a preliminary conclusion, as only eight carcinogens were considered so far. Furthermore, an unfavorable health impact related to HTPs remains as compared to complete abstinence. Our method results in useful information that may help policy makers in better understanding the potential health impact of new tobacco and related products. A similar approach can be used to compare the carcinogenicity of other mixtures.

## 1 INTRODUCTION

Many new types of tobacco product have emerged on the market in recent years, the most prominent examples being electronic cigarettes and heated tobacco products (HTPs) (El‐Toukhy et al., 2018; Elias, Dutra, St. Helen, & Ling, 2018; Hair et al., 2018; Kim, Yu, Lee, & Paek, 2018; Nyman et al., 2018; Tabuchi et al., 2018). Many of these new tobacco and related products (TRPs) claim to be “harm reduction” or “reduced risk” products (Martin et al., 2018). Some jurisdictions include provisions regarding modified risk products in their laws. For instance, the U.S. Family Smoking Prevention and Tobacco Control Act defines a “Modified risk tobacco product as a tobacco product sold or distributed to reduce harm or reduce the risk of tobacco‐related disease associated with commercially marketed tobacco products.” An example of an HTP evaluated as modified risk product is the IQOS device that is used to heat tobacco sticks (HEETS) (FDA, 2017). HEETS, containing reconstituted tobacco and additives such as nicotine and flavorings, are heated with an IQOS. The resulting emissions, which are inhaled by the user, are claimed to contain less harmful compounds and should therefore be less hazardous to human health than conventional cigarettes.

According to the World Health Organization (WHO) (WHO, 2014), regulators need to assess the validity of implicit or explicit health claims of new TRPs for the individual user by evaluating various criteria. One of those is that, compared to a reference product, the new TRP needs to have lower emissions or toxicants levels in mainstream smoke, lower overall toxicant exposure, and fewer detrimental effects at the individual level. In addition, the potential impact on public health at the population level needs to be estimated by assessing the total prevalence of all TRP users, dual users of TRPs and cigarettes, and original TRP users switching to cigarette smoking (“gateway” effect).

Establishing the health impact of smoking cigarettes or alternative tobacco products is a complex issue. Novel tobacco products often show reduced emissions but it remains unclear how this translates to reduction of cigarette smoke‐induced health effects. Cigarette smoke is a complex mixture containing more than 7,000 compounds with varying toxicological effects, at least 63 of which may cause cancer (USDHHS, 2014). In addition to different types of effect, there is also the issue of varying potency (the dose at which effects start to occur) and varying severity (e.g., cancer vs. mild lung lesions). Several methods for hazard and/or risk prioritization of compounds in cigarette smoke have been proposed (Burns et al., 2008; Cunningham, Fiebelkorn, Johnson, & Meredith, 2011; Fowles & Dybing, 2003; Lachenmeier & Rehm, 2015; Pankow, Watanabe, Toccalino, Luo, & Austin, 2007; Xie et al., 2012). Stephens (2017) proposed a method for comparing the potential risks of alternative products relative to that of cigarettes by using the inhalation unit risk, also called cancer potency factor (CPF). While his general approach of taking the sum of the potency‐adjusted emissions is valid, inhalation unit risks suffer from various drawbacks as acknowledged in his paper. For example, they are determined in a nonstandardized way, and, as a result, they can be expected to result in an imprecise estimate of the potency of each constituent compound in the product, and hence of the product as a whole. This is discussed in more detail below, and illustrated by some results (Appendix C in Supporting Information). Another way of estimating relative potencies is by applying the benchmark dose (BMD) approach, which has a solid statistical foundation, and relies on the dose‐response data rather than on simply assuming that the dose response is linear starting from a more or less coincidental reference point. Furthermore, it is important to take the quantitative uncertainties in the underlying data (both on exposure and on dose response) into account, and explicitly quantify the ensuing uncertainty of the outcome, without which unwarranted decisions might be taken by policy makers.

We have developed a method that uses relative potency estimates based on dose‐response modeling and evaluates the uncertainties in the underlying data. The resulting change in cumulative exposure (CCE) may be used to assess the impact on health effects in a semiquantitative way. Before providing a more extensive discussion of the method by an application to a case study, we will first describe the rationale of the approach in conceptual terms.

## 2 CONCEPTUAL SUMMARY OF THE METHOD

The method we propose relies on the core method of dose addition, a method frequently applied to assessing the risk of combined exposures to multiple compounds (Meek et al., 2011). It assumes that the dose responses (for the same type of effect) of the compounds involved are parallel (on log‐dose scale). This assumption appears to be generally valid (EUROMIX, 2017), and can be evaluated in retrospect by examining the results of the dose‐response analysis (see Supplementary Material, SM2). It might be that some of the compounds in tobacco smoke deviate from dose addition and are subject to synergism or antagonism. However, interaction among chemicals seems to be rare (EUROMIX, 2017), and even if it occurred, the ensuing under‐ or overestimating of risks would occur in both products that are being compared. Since we quantify the ratio of the cumulative exposures in each product, over‐ or underestimations due to interaction would at least partly cancel out (see expression (1)).

**Fig. 1:**
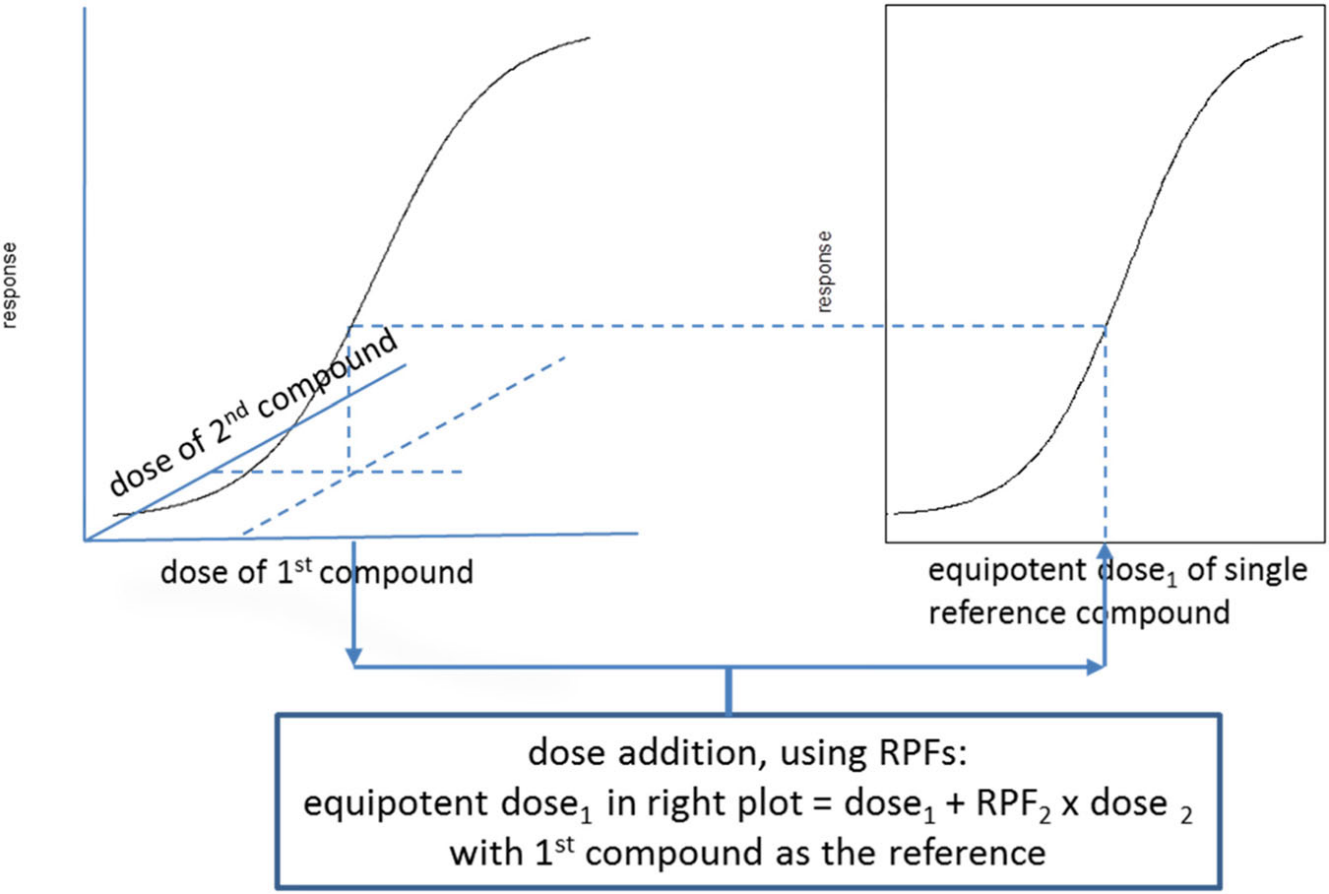
Schematic representation of the concept of dose addition. The multiple doses of the mixtures (left plot; only two compounds shown here in this three‐dimensional figure; in reality, there is a response surface rather than a curve) are translated into an equipotent dose of one of the constituent compounds, called the reference compound (right plot). Note that the dose‐response curve in these plots may be unknown; nonetheless, we can assume that the response at the mixture dose will be similar to that of the single dose of the reference compound.

Dose addition involves expressing the dose of the mixture, consisting of constituent compounds each with its own dose, as an equipotent dose of one of the constituent compounds (called the reference compound). For a schematic illustration, see Fig. 1. Using this method, we can transform the problem of having a tobacco product with multiple compounds to a hypothetical product that only contains the reference compound with a dose (emission) that would result in the same effect as the mixture in the real tobacco product. In this way, the difficulty of trying to find the response to multiple compounds in the tobacco product is reduced to that of finding the response of a single dose related to a single compound. The latter single dose is termed cumulative exposure below.

A second aspect of reducing the problem into more tractable terms is by considering the ratio of the cumulative exposure in one product (e.g., HTP) relative to that of another product (e.g., combustible cigarette). This ratio expresses the CCE between both products. As a first advantage, the CCE does not depend on the choice of the reference compound. The great advantage of the CCE is that for making inferences on the approximate health impact of switching between tobacco products we can make inferences directly based on human dose‐response information related to cigarette smoking, as discussed below (see Step 6). In this way, the extrapolation of animal dose response into human dose response, with its associated uncertainties, can be avoided. The assumption needed here is that the relative potency factors (RPFs), estimated from animal data, are a reasonable approximation of the RPFs in humans. In risk assessments of mixtures based on dose addition, this assumption is always implicitly made.

The third aspect of the method is the evaluation of uncertainties in the underlying data, due to limitations or other weaknesses in the available data. These are uncertainties in the estimated emissions of the constituent compounds (due to limitations in the measurements), as well as the uncertainties in the estimated RPFs (due to limitations in the dose‐response data). The uncertainties are translated in the ensuing uncertainty in the final outcome: the estimated CCE. To illustrate the importance of knowing the latter uncertainty, imagine that the CCE is estimated to be a 20‐fold decrease in cumulative exposure for a given novel tobacco product.

However, when the evaluation of the uncertainty in the value resulted in a range from 1.3‐fold to 120‐fold, there would not be much ground for decision‐making based on the point estimate of 20‐fold: a quite small CCE of 30% cannot be excluded. If, on the other hand, the uncertainty range was found to be between 10‐fold and 40‐fold, then this would constitute support that the alternative product does indeed lead to a (substantial) favorable health impact.

**Fig. 2:**
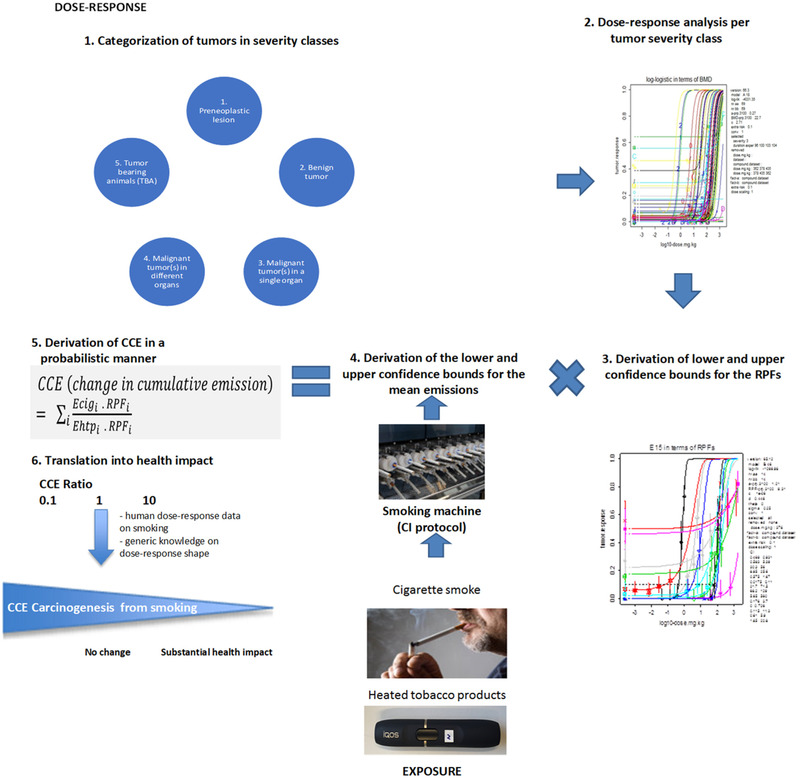
Graphical illustration of CCE derivation (CCE = change in cumulative emission; *E_cig_* = cigarette emissions; *E_htp_* = heated tobacco product [or alternative tobacco product] emissions; RPF = relative potency factor). Step 1) Categorization of tumors in severity classes: for each cancer study, observed lesions were categorized into either preneoplastic lesion, benign tumor(s), malignant tumor(s) in a single organ, malignant tumor(s) metastasizing in different organs, or tumor‐bearing animals as suggested by an experienced pathologist. Step 2) For each tumor severity category, dose‐response analysis was performed for each individual data set (same tissue, sex, tumor type, exposure duration, and study duration). Step 3) For each tumor severity category, a critical data set for each compound was selected based on the lowest BMD estimate; BMD analysis was performed on the combined critical data sets (one per compound), resulting in confidence intervals for the RPF. The results for the various severity categories were combined, as described in the final part of Supplementary Material SM2. Step 4) Determination of confidence intervals for mean emissions, based on data from smoking machine studies. Step 5) The change in cumulative emission (CCE) was derived by taking the uncertainty in the RPF and the emissions between cigarettes and HTPs into account, resulting in an uncertainty range for the CCE. Step 6) The value (uncertainty range) of the CCE is translated into an estimate of the health impact, based on available dose‐response data of smokers, and generic knowledge on the steepness of dose responses in general. A CCE of 1 would be associated with no change in health impact, while a factor of 10 or more may be expected to result in a substantial reduction in harm, when a user switches from cigarettes to HTP. A CCE (substantially) smaller than 1 would indicate an increase in harm.

The method is graphically summarized in Fig. 2 in six separate steps.

## 3 ILLUSTRATIVE CASE STUDY

To illustrate how the method works in a practical analysis, it was applied to eight carcinogens common to both HTPs and cigarettes, for which both emission and cancer dose‐response data were available. This section discusses the six steps involved in the method in somewhat more detail. More details can be found in the Supplementary Material.

### 3.1 Step 1: Carcinogenicity Data and Severity Categories

Carcinogenicity data were obtained from the carcinogenic potency database (CPD, 2018) and from the US National Toxicology Program database (NTP, 2018). In addition to the eight compounds directly relevant for the evaluation, the dose‐response analyses in steps 2 and 3 (see below) were performed using an additional nine compounds with inhalation carcinogenicity data for an exposure period longer than 96 weeks. These nine compounds were additionally included in the BMD analysis to reduce the uncertainty in the estimated RPFs, due to a more precise estimate of the steepness parameter in the dose‐response model when more compounds provide information on that value (as a sort of read‐across; see Slob & Setzer, 2014, p. 288, for an explanation of this principle). All compounds used in the dose‐response analyses are listed in Table I.

**Table I:** Inventory of Compounds with Emission, Carcinogenicity, and Inhalation Unit Risk Data

Compound	Abbreviation	IARC category	Emission data (CS and HTP)	Carcinogenicity data	Inhalation unit risk data
Acrylonitrile	can	2B	√	√	√
Acetaldehyde^a^	ald	2B	√	√	√
1,3‐Butadiene^a^	but	1	√	√	√
Ethylene oxide	eox	1	√	√	√
Formaldehyde^a^	fal	1	√	√	√
Benzo[a]pyrene^a^	bap	1	√	√	√
Nitrobenzene	nbz	2B	√	√	√
Propylene oxide	prp	2B	√	√	√
Allyl glycidyl ether	age	NC		√	
Alpha‐methyl styrene	ams	2B		√	
1,2‐Dibromo‐3‐chloropropane	dbcp	2B		√	√
1,2‐Dibromoethane	dbe	2A		√	√
Decalin	dcn	NC		√	
Hydrazine	hyr	2A		√	√
Isobutyl nitrite	isn	2B		√	√
Naphthalene	nap	2B		√	√
Propylene glycol mono‐t‐butyl ether	pge	2B		√	

^a^WHO TobReg list of smoke components for mandated lowering (Burns et al., 2008). CS = cigarette smoke; HTP = heated tobacco product; NC = not classified by IARC.

Severity categories were assigned to each measured endpoint because cancer dose‐response data may relate to distinct endpoints, reflecting different stages of the carcinogenicity process, related to varying degrees of severity. Clearly, a dose that causes a 10% increase in the fraction of animals with a preneoplastic lesion such as hyperplasia is not equipotent to a dose that causes a 10% increase in animals with malignant carcinomas. We assigned one of the following “lesion categories” to each endpoint in our database: (1) preneoplastic lesion, (2) benign tumor(s), (3) malignant tumor(s) in a single organ, (4) malignant (metastasizing) tumors in different organs, and (5) tumor bearing animals (Hernandez, van Benthem, & Slob, 2012). These assignments were based on a full list of possible endpoints in cancer studies, compiled by an experienced pathologist. Steps 2 and 3 below were performed for each severity categories 2–5 separately.

### 3.2 Step 2: Selection of Data Sets with the Lowest BMD for Each Compound

The BMD software PROAST (see www.proast.nl) version 65.12 was used for dose‐response analysis. In this step, the dose‐response analysis was performed to determine the individual data set with the lowest BMD estimate for each compound. An individual data set was defined as one where all associated factors were at the same level (same compound, tissue, sex, tumor type, exposure duration, study duration). The results of this first dose‐response analysis are shown in SM2.

### 3.3 Step 3: Estimation of RPFs

In Step 3, the critical data sets (for each severity category) were combined for BMD analysis to estimate the RPF for each compound. The dose‐response curves for the different compounds can be described by parallel dose‐response curves (on log‐dose scale), and the distance between each pair of these curves is the log of the RPF. The RPF can be estimated by the ratio of the BMDs for both chemicals. Due to the curves being parallel on log‐dose scale, the BMD ratio does not depend on the value of the BMR (note that a ratio turns into a difference or distance on log‐scale). 1,3‐Butadiene (but) was selected as a reference compound because dose‐response data were available for all severity categories 2–5. The reason for choosing the same reference compound for each severity category was that BMDs might systematically differ among these categories, whereas estimated RPFs might be less dependent on category. An exponential model (see SM2) was used for estimating the RPF for each of severity categories 2–5. This resulted in RPFs for 9, 13, 4, and 13 compounds related to severity categories 2–5, respectively. These were translated into a single RPF confidence interval for each compound (see SM2), resulting in the confidence intervals shown in Table II.

**Table II:** Confidence Intervals of the RPFs of Each Compound Relative to 1,3‐Butadiene, Estimated from BMD Analysis of the Dose‐Response Data Available for Inhalation Carcinogenicity Studies. See SM2 for More Detailed Data Per Severity Category, and How They Were Integrated into One Single Confidence Interval for Each Compound

Substance	LB	UB
1,3‐Butadiene ^*^ ^a^(BMD)	30.3	56
Acrylonitrile^a^	5.76	192
Allyl glycidyl ether	0.089	2.69
Acetaldehyde^a^	0.469	0.931
Alpha‐methyl styrene	0.583	5.39
Benzo[a]pyrene^a^	29.3	187
1,2‐Dibromo‐3‐chloropropane	8.95	26.6
1,2‐Dibromoethane	0.575	1.67
Decalin	0.0175	0.11
Ethylene oxide^a^	21.7	71.5
Formaldehyde ^$^	68.2	129
Hydrazine	5.65	560
Isobutyl nitrite	0.176	3.7
Naphthalene	0.339	0.726
Nitrobenzene^a^	0.115	11.3
Propylene glycol mono‐t‐butyl ether	0.61	5.9
Propylene oxide^a^	1.85	22.8

^a^Emission data also available; LB = 5% lower uncertainty bound; UB = 95% upper uncertainty bound.

### 3.4 Step 4: Evaluation of Emission Data

An inventory was made up of smoke components measured in HTPs and in conventional cigarettes (Auer, Concha‐Lozano, Jacot‐Sadowski, Cornuz, & Berthet, 2017; Bekki, Inaba, Uchiyama, & Kunugita, 2017; Jaccard et al., 2017; Li et al., 2019; Mallock et al., 2018; Schaller, Pijnenburg, Ajithkumar, & Tricker, 2016; Uchiyama et al., 2018). As we are interested in the relative change in emissions between HTP and cigarette, we selected the study by Schaller, Pijnenburg, et al. (2016), as they measured the emissions under similar circumstances in both products, using similar assumptions on smoking behavior. When there is knowledge about different smoking behaviors in both products (e.g., # puffs per minute is larger in one product), this can be taken into account in our method, as illustrated in Appendix A of Supporting Information.

The concentrations in Schaller, Keller, et al. (2016) were compared to various other publications reporting HTP emission data (Jaccard et al., 2017; Li et al., 2019; Mallock et al., 2018; Uchiyama et al., 2018), to make sure that the concentrations mentioned in this publication were in the same range. Some differences were found, which might be explained by the different smoking regimes used; however, it is not the emissions themselves but the ratio between emissions that affects the estimated value of CCE. As long as both products were measured in the same and relevant way, the ratio of the measurements should provide the appropriate information.

Table III shows the uncertainty in the mean emissions for any of the two products expressed as (two‐sided) 90% confidence intervals based on replicate measurements (see SM2).

**Table III:** Uncertainty Bounds for the (Geometric) Mean Emissions Per Stick (HTP) or Per Cigarette Based on Data from Schaller, Keller, et al. (2016)

	HTP Emission (μg/stick)		Cigarette Emission (μg/cigarette)	
Compound	LB	UB	LB	UB
Acrylonitrile	0.142	0.320	30.82	32.71
Acetaldehyde	143	321	1,048	2,358
Benzo[a]pyrene	0.00092	0.00207	0.0093	0.0209
1,3‐Butadiene	0.212	0.477	51.02	114.79
Ethylene oxide	0.162	0.364	21.14	47.56
Formaldehyde	3.560	8.010	41.53	93.45
Nitrobenzene	0.00006	0.00051	0.00015	0.0327
Propylene oxide	0.102	0.229	1.005	2.260

HTP = heated tobacco product; LB = 5% lower uncertainty bound; UB = 95% upper uncertainty bound.

### 3.5 Step 5: Derivation of Change in Cumulative Emission

The cumulative emission of the eight compounds for both cigarette and HTP was derived taking into account the RPF (relative to 1,3‐butadiene) for each compound. This resulted in an estimate of the cumulative emission, expressed in amounts of 1,3‐butadiene. The ratio of the two estimates reflects the change in cumulative emission of the alternative product (e.g., HTP) as compared to the cigarette:

(1) $$\begin{array}{ccc} & & CCE\;=\;change\;in\;cumulated\;emission\;=\\ & & \;\frac{\sum_{i}Ecig_{i\;}\;.\;RPF_{i}}{\sum_{i}Ehtp_{i\;}\;.\;RPF_{i}} \end{array}$$

where the subscript *i* denotes a particular compound, and *E_cig_* and *E_htp_* the emission in μg/cigarette and in μg/HTP stick, respectively. A value of CCE larger than 1 indicates a decrease in cumulative emission relative to cigarette smoke, and vice versa. It should be noted that selecting another reference compound would result in the same estimate of the CCE.

We combined the confidence intervals for the emissions of the eight compounds (in cigarette and in HTPs) with the confidence intervals of the RPFs, and used that in a probabilistic approach to calculate the overall uncertainty in the estimated CCE. The probabilistic evaluation is done by defining a (lognormal) distribution for *E_cig_*, *E_rpf_*, and RPF, based on their lower and upper (two‐sided) 90% confidence bounds, reported in Tables II and III. Then, for each of these three distributions, a large number (*n*) of values is sampled and for each combination of the three randomly sampled values, the expression is evaluated, resulting in *n* values for CCE. Together, these values constitute the uncertainty distribution for CCE, the 5th and 95th percentiles of which define a two‐sided 90% confidence interval for the CCE.

As shown in Table I, there were eight compounds for which both emission data and RPF estimates were available. Based on these eight compounds, the probabilistic evaluation of the CCE (see expression (1)) resulted in an uncertainty range of 9.6–26. In other words, the cumulative emission for this eight‐compound mixture is estimated to be somewhere between 10‐ and 25‐fold lower (after rounding off), when a given individual consumes the same number of HTPs instead of cigarettes (with similar smoking behavior for both products).

### 3.6 Step 6: Translating CCE Into Health

The final question to be answered is what (individual) health impact may be associated with a decrease in (cumulative) exposure by a factor somewhere between 10 and 25. As one of the advantages of the proposed method, this 10‐ to 25‐fold CCE relates to the whole mixture (considered), or, equivalently, to any single compound used as a reference (see Fig. 1). Therefore, we can use our generic knowledge about the typical steepness of a toxicological dose‐response relationship for a single compound to make inferences on the associated health impact. Based on the steepness of a dose response, it can be inferred whether a decrease in dose by 10‐ or 25‐fold will lead to a minor or a substantial reduction of the effect. However, quantal dose‐response relationships (describing incidence as a function of dose) are not suitable for that purpose, as they will be steeper in animals than in humans, because animals in toxicity studies are much more homogeneous than humans. Continuous dose‐response relationships are not sensitive to interindividual variation and there is no reason that they would differ in shape among animals, including humans. This is supported by a reanalysis of a large number or dose‐response data sets from rats and mice (Bokkers & Slob, 2007). From general experience and systematic reviews (Slob & Setzer, 2014), it is known that dose responses related to continuous endpoints are in general quite homogenous regarding their steepness: the ratio between BMD10 and BMD05 (doses associated with a 10% and a 5% effect size) is usually close to, or less than 2. For the time‐to‐tumor dose response reported in a mega‐rat study (Peto, Gray, Brantom, & Grasso, 1991), the BMD10/BMD05 ratio was found to have a confidence interval of 2 up to 2.6 (Slob & Setzer, 2014). So, in the rat, a CCE of 2 to 2.6 would reduce a 10% reduction in life span to a 5% reduction. This implies that a CCE between 10 and 25 should be associated with an even greater (favorable) impact on life span.

Another approach for relating a CCE with health impact is to use dose‐response information on smokers. For example, a recent study (Inoue‐Choi et al., 2018) compared nondaily to daily smokers (50 vs. 600 cigarettes per month) and found that life expectancy for both groups was reduced by 5 and 10 years, respectively. The difference in exposure to cigarette smoke (i.e., just over 10 fold) was apparently associated with a five‐year longer expected life span. As this difference in exposure between the nondaily and daily smokers is comparable to the (lower bound) CCE value of 10‐fold between HTP and cigarette, it may be inferred that the associated health impact of that CCE is substantial. At the same time, nondaily smokers were reported to have a five‐year shorter life span compared to nonsmokers, and this indicates that HTP would still be associated with a substantial (∼five years) decrease in life span.

While the study in rats indicated that a reduction in healthy life span from 10% to 5% would be associated with a decrease in dose by 2‐ to 2.6‐fold, the study on smokers related a roughly similar change in life span to a decrease in exposure by a factor of around 10. This seems to contradict the assumption just made that (continuous) dose responses are equally steep among species. However, this might be explained by the fact that the observed dose response on life expectancy in the (non)daily smokers integrates various types of effect, including cancer and cardiovascular effects, which may lead to less steep dose response for overall life expectancy.

Overall, the conclusion seems to be warranted that consuming HTPs instead of cigarettes will be associated with a substantial increase in life expectancy, for the subgroup of smokers who would die from cancer. However, a substantial negative health impact is expected to remain from consuming HTPs as compared to total abstinence from tobacco products.

## 4 DISCUSSION

We have developed a methodology for evaluating the potential magnitude of the health impact when comparing a cigarette smoker with a consumer of an alternative tobacco product (with similar consumption pattern; see Appendix A of Supporting Information if consumption pattern is known to be different). The method we propose here was applied to cancer, but we intend to examine whether it can be adapted for other types of effect (e.g., cardiovascular) that may be caused by the use of tobacco products. In addition, the method can also be applied to evaluate the carcinogenicity of other complex mixtures. Our method does not consider the potential impact of the alternative product on public health at the population level. The required information (e.g., total prevalence of all TRP users, dual users of TRPs and cigarettes, and original TRP users switching to cigarette smoking) is not available when a new TRP is launched on the market, and can only be roughly estimated by information from other markets, similar products, or estimated product appeal for several relevant user groups.

Estimating cancer risk (as one potential type of effect that may be caused by tobacco use) by applying current risk assessment methods to each single component of the mixture is an almost impossible undertaking because of the large number of compounds present in cigarette smoke, and because current methods aim at safety assessment rather than estimating health effects. Furthermore, we found that inhalation unit risk values (one of the current methods of cancer risk assessment) are not (or barely) informative regarding the (relative) potencies of the compounds (see Appendix C of Supporting Information). Instead, we estimated the relative potencies of the compounds based on dose‐response modeling, which is the (statistically) appropriate method for that purpose. By focusing on the estimation of the CCE, we circumvent various difficulties that would occur when applying current risk assessment methods to each individual compound. It is much easier to draw conclusions on health impact based on the *CCE* relative to the cigarette, as shown in the discussion of Step 6 of the case study. The main assumption in our calculation of the CCE is that the relative potencies among compounds estimated from animal data approximately hold for humans. Based on the CCE, the health impact can be inferred from general dose‐response information on the steepness of dose‐response curves in general, or from available dose‐response information in smokers with various use intensities. Thus, we found that the CCE most likely amounts to a factor between 10 and 25 when comparing a leading variant of HTPs with cigarettes, and that even the lower bound of this uncertainty range would be associated with a substantial health impact in favor of HTP. Obviously, the health impact will be greatest for habitual smokers when they switch to HTP at a young age.

### 4.1 Some Advantages of the Method

A major advantage of our approach is that it makes the uncertainty in the final estimate of the CCE visible, which is not possible in approaches based on inhalation unit risk. As the probabilistic calculation of the CCE showed, the uncertainty in that value (range of 10‐ to 25‐fold) in this particular case study is relatively small, and allows for the conclusion that the reduction in cumulative emission is substantial, even for the lower bound of the CCE estimate. However, in other cases, e.g., when data are more limited for some of the compounds, the estimated CCE may be much more uncertain. In those cases, a single point value, without the uncertainty bounds, may indeed be misleading, and inaccurate conclusions can be easily drawn.

A specific point in Steps 2 and 3 is that we applied dose‐response analysis to the combined data sets available in a single model fit. This method allows for an estimate of the RPF for data‐poor carcinogens, or carcinogens with no significant increase in incidence with dose; this is possible by applying the covariate approach in the dose‐response analysis (Soeteman‐Hernandez, Johnson, & Slob, 2016). Such poor or weak data sets may have resulted in RPF confidence intervals with lower bound = 0 and upper bound = infinite, i.e., totally noninformative. In the covariate approach, even data sets with a nonsignificant trend will result in a useful RPF confidence interval (with 0 lower bound but with finite upper bound), and that uncertainty can be evaluated in the probabilistic evaluation of the CCE. In this way, the uncertainty of such poor data sets is propagated into the uncertainty of the final CCE. Note that very small values of the RPF will hardly change the value of the CCE.

### 4.2 Number of Compounds Considered

Our analysis is based on only eight carcinogenic smoke components, while many more carcinogens are present in smoke. However, if it were assumed that these eight compounds are a representative sample of all carcinogens occurring in smoke, then increasing the number of compounds in the analysis will make the estimate of the CCE more reliable, but most likely not dramatically different. Nonetheless, it would be better to include more carcinogens in the analysis when the data are available. For instance, some compounds might show higher rather than lower emissions in HTP, which could reduce the value of CCE. That such is possible was shown by St. Helen, Jacob Iii, Nardone, and Benowitz (2018) who claimed that 22 substances were threefold higher and seven were >10‐fold higher in the HTP (IQOS) than in 3R4F reference cigarette smoke. As long as that information is not incorporated in an analysis that estimates the CCE, it remains unclear how those compounds may influence the CCE and the associated health impact. When they happen to have a relative high RPF, they may reduce the value of the CCE, but if their RPFs are relatively small, they will not.

### 4.3 Carcinogenicity Dose‐Response Limitations

While we have tried to obtain the best possible estimates of relative potency among compounds, by using the most recent BMD methodology, and by taking biological factors into account to our best ability, there are some remaining issues in the dose‐response analysis that need to be mentioned. First, all study durations longer than 96 weeks were considered similar, while it is known that study duration may have a considerable impact on the dose response for cancer endpoints (see, e.g., the mega‐mouse study with 2‐acetylaminofluorine [Ittrich et al., 2003]). To further improve the estimation of RPFs, dose‐response models should be developed that can take study duration into account in an efficient manner. Given the current state of the science, our approach was to assume no differences in study duration. Fortunately, the majority of the data sets were related to study durations around 100 weeks with only a few in the range of 120 or 150 weeks. Therefore, the bias in the RPF estimates may be limited. Another issue is that the RPFs were based on data sets with largely different background response. We used extra risk as a way to correct for background response, as this is the default way of expressing the BMR in most international organizations (e.g., EPA [USEPA, 2012], EFSA [EFSA, 2017], WHO [1994], [WHO‐IPCS, 2014]). However, the problem is that it is not entirely clear how to properly adjust for background response (WHO‐IPCS, 2014). This problem may also lead to bias in some of the RPF estimates. Yet, it seems unlikely that it will have a substantial impact on the overall estimate of the CCE.

### 4.4 Using Alternative Data

As carcinogenicity studies are currently only performed when considered indispensable, alternative approaches of estimating RPFs for carcinogenic compounds need to be explored. One promising avenue is the correlation found between the BMDs derived from *in vivo* micronucleus tests (i.e., a short‐term genotoxicity test that measures chromosomal aberrations) and BMDs from carcinogenicity studies (Bemis et al., 2016; Johnson et al., 2014, 2014; Soeteman‐Hernandez, Fellows, Slob, & Johnson, 2015; Soeteman‐Hernandez et al., 2016; Wills, Johnson, Battaion, Slob, & White, 2017; Wills, Johnson, et al., 2016; Wills, Long, et al., 2016). Given this correlation, it directly follows that data from the *in vivo* micronucleus test might be a suitable surrogate for estimating RPFs of carcinogens. Regarding nongenotoxic carcinogens, a possible option is to use subchronic No‐Observed Adverse Effect Level (NOAELs) (or preferably, BMDLs) because they have been shown to correlate well with the BMDs from carcinogenicity studies (Braakhuis et al., 2018).

### 4.5 Implications for Policy and Practice

Our method may have significant implications for policy and practice, as it provides a tool to evaluate the health impact of an individual who switches from smoking cigarettes to new TRPs.

An outcome of the method is an uncertainty range for the CCE, which is informative by itself at least for comparison among various new TRPs. By making the uncertainty in the CCE visible for any specific case, policy makers know if available scientific information is weak or sufficiently strong to provide a basis for their decisions. Furthermore, the potential health impact associated with specific values of the CCE can be estimated based on general information on the steepness of toxicological dose‐response relationships in general, or on more specific dose‐response information on smokers.

## Supporting information


**Figure B1**. Uncertainty ranges of the RPF‐adjusted emissions (expressed as butadiene equivalents) of eight carcinogenic compounds in cigarette (dashed lines) and in HTP emissions (solid lines).
**Figure C1** Inhalation unit risks (also called cancer potency factors [CPFs]) plotted against RPFs both on a log scale.

Supplementary Material

Supplementary Material
