# Supplementary material for: A Method for Comparing the Impact on Carcinogenicity of Tobacco Products: A Case Study on Heated Tobacco Versus Cigarettes
Source: Risk Anal. 2020 May 1;40(7):1355–66. doi: 10.1111/risa.13482 (PMC7496151; doi:10.1111/risa.13482)
Supplement: Supplementary file 1 — Figure B1. Uncertainty ranges of the RPF‐adjusted emissions (expressed as butadiene equivalents) of eight carcinogenic compounds in cigarette (dashed lines) and in HTP emissions (solid lines). Figure C1 Inhalation unit risks (also called cancer potency factors [CPFs]) plotted against RPFs both on a log scale. [file RISA-40-1355-s001.docx]

**Appendix A. Adjusting the CCE for exposure conditions**

Various assumptions are required to estimate values for the CCE based on measurements of emissions that are representative of exposure. First, it is assumed that the relative differences in emissions between cigarette and HTP as reported by Schaller et al. (Schaller, Pijnenburg, Ajithkumar, & Tricker, 2016) adequately reflect the (fold-)change in exposure to the measured compounds when a given smoker consumes one cigarette or one HTP. This will be the case when the puff regime (puff volume, puff duration, inter-puff interval, number of puffs per minute, and total puff volume) does not change from consuming one HTP item versus one cigarette. Further, it must be assumed that the HTP-user would consume the same number of sticks per day (in the long run) as cigarettes. If information is available on changes in these factors, they can be easily incorporated into the formula of the CCE. As an illustrative example, suppose it is known that changing from cigarette to HTP would result in a lower relative smoking rate (RSR) by 30%, then CCE can be multiplied by 1.42 (= 1/0.7; note that such adjusting factors need to be expressed as a fold change, not as a percentage). When it is assumed that the RSR holds both for heavy and light smokers, then the adjusted CCE also holds for smokers of different smoking intensity when they change from cigarettes to HTP. If the value of CCE is only known approximately, that uncertainty can be taken into account be probabilistically by evaluating the expression (equation A1):

$CCE=RSR \frac{\sum_{i} {Ecig}_{i} . {RPF}_{i}}{\sum_{i} {Ehtp}_{i} . {RPF}_{i}}$

by assuming an uncertainty distribution for RSR (in particular lognormal), such that the 5^th^ and 95^th^ percentiles reflect the assumed uncertainty bounds in the RSR (expressed as a fold change). Similarly, other factors that might have an impact on smoking behavior may be expressed as a fold-change in relative exposure between cigarette and HTP, which can be added to expression (2). Note that information on the absolute levels of these smoking behavior factors is not needed, as the CCE only expresses a relative change for a given individual.

**Appendix B. Ranking RPF-adjusted emissions**

Figure B1 shows the uncertainty ranges of the RPF-adjusted emissions for each individual compound, both for cigarette and for HTP. These results can be used for ranking the compounds. For example, the contribution of *fal* to the cumulative emission is larger than that of *but*, both in cigarettes and in HTPs, However, no ranking is possible for *acn* and *prp*, as their uncertainty ranges (for HTPs) overlap. Given these uncertainty ranges it is however hardly possible to conclude for which compounds the RPF-adjusted emission decreased most in HTP relative to cigarette. The uncertainty ranges in Fig. 2 were obtained by multiplying the confidence bounds of emissions and RPFs with each other, and therefore should be considered as conservative uncertainty ranges. These uncertainty ranges could also be established using a probabilistic approach, leading to slightly smaller uncertainty ranges.


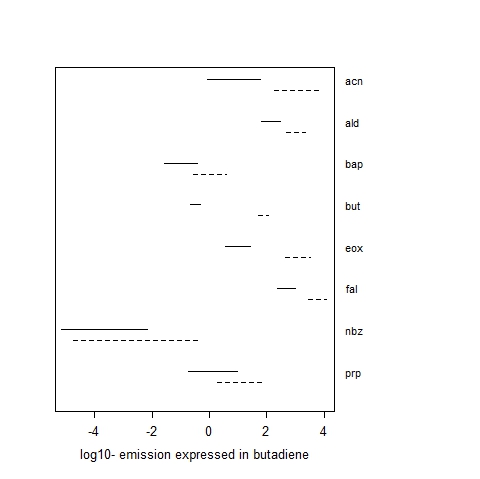


**Figure B1.** Uncertainty ranges of the RPF-adjusted emissions (expressed as butadiene equivalents) of eight carcinogenic compounds in cigarette (dashed lines) and in HTP emissions (solid lines). Only non-overlapping ranges allow for ranking.

**Appendix C. Correlation between unit risk and RPF**

Our proposed method is similar to an earlier proposed approach (Fowles & Dybing, 2003; Stephens, 2017) that used inhalation unit risks values, also called cancer potency factors (CPFs). The inhalation unit risk is the upper-bound estimate of the excess lifetime cancer risk resulting from continuous exposure to an agent at a concentration of 1 µg/m^3^ in air. The inhalation unit risk is often used for setting air guideline values, which are concentrations of compounds in air that are associated with an acceptably small risk in the human population (Stephens, 2017; WHO, 1994, 2000a, 2000b). The estimation of inhalation unit risk involves the extrapolation from dose levels with relatively high (observable) risk, to dose levels with very low (acceptable, but non-observable) risk. A common extrapolation model used is the linearized multistage (LMS) model, based on the assumption that this model reflects the mainlines of the mechanisms of carcinogenesis (WHO, 2000a). Another method is linear extrapolation, which assumes that the dose-response is linear between a chosen point of departure and dose zero. These methods are based on assumptions on the dose-response in the non-observable region, and the results heavily depend on those assumptions, while it is unclear whether these assumptions are valid. Another drawback of unit risk values is that they are not derived in a consistent manner. For example, the uncertainty in the point-of-departure (PoD) may or may not be taken into account, the underlying data do not consistently relate to the same species, and varying additional assessment factors are used. In some cases, linear extrapolation is used while the data are not in line with that assumption. In addition, the unit risk precludes the possibility of evaluating the precision of the associated value.

Despite these weaknesses, inhalation unit risk values might be good enough for getting an approximate value of the CCE, by inserting them into expression (1) instead of the RPFs, as a first tier assessment. To investigate this option, we collected inhalation unit risk values for the compounds we analyzed for obtaining RPFs (see Table 1). We used various sources, including the Office of Environmental Health Hazard Assessment database (OEHHA, Cal EPA, California, USA), the US Environmental Protection Agency’s Integrated Risk Information System (US EPA IRIS) (IRIS, 2017), Fowles and Dybing (Fowles & Dybing, 2003), Talhout et al. (Talhout et al., 2011), O’Brien et al (O'Brien, 1996), ACCAP (ACCAP, 2003), the Department of Environment Quality (DEQ), and the Texas Commission on Environment Quality (TCEQ) (TCEQ, 2015). Thus, data for inhalation unit risks were found for 13 out of the 17 compounds (Table 1).

Plotting the RPFs against the inhalation unit risks showed very poor or no correlation between these two parameters (Fig. C1), even though they ranged over several orders of magnitude. Also of interest is that the unit risks ranged over around four orders of magnitude, while the range in RPFs is close to two orders of magnitude. The larger range in unit risks can be explained by the variation in the way they have been established, as just discussed. This result precludes the option of calculating the CCE based on unit risks in a first tier assessment. It would result in unreliable results, and unfortunately the much more elaborate estimation of RPFs based on dose-response analysis cannot be avoided.


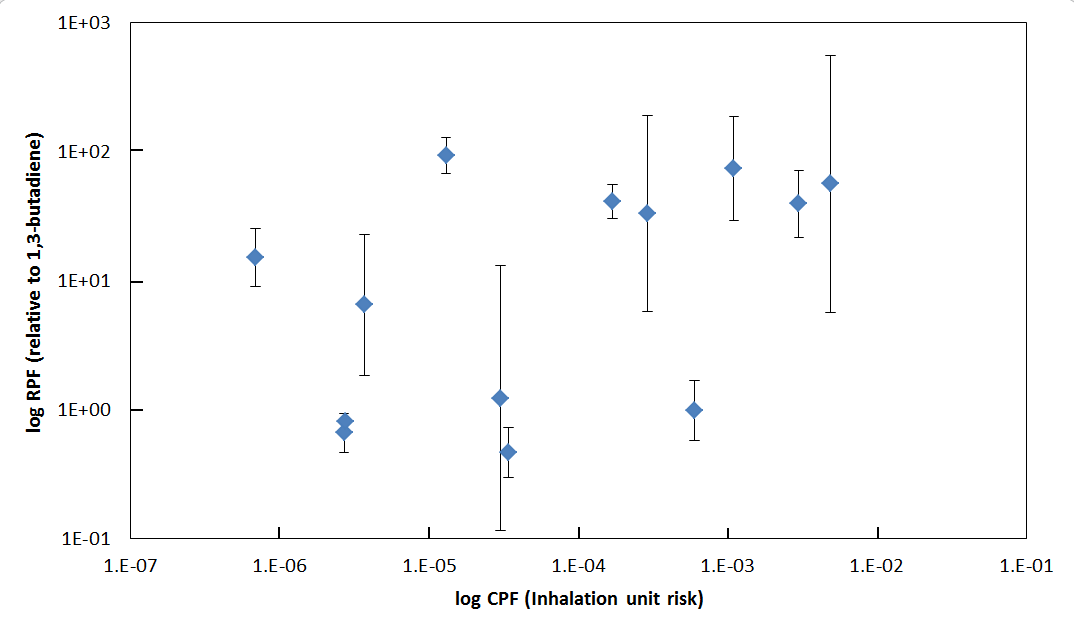


**Figure C1** Inhalation unit risks (also called cancer potency factors, CPFs) plotted against RPFs, both on a log-scale. Confidence intervals are plotted only for the RPFs as inhalation unit risks do not allow for their calculation.

ACCAP. (2003). American Chemistry Council Acetone Panel, Voluntary Children’s Chemical Evaluation Program. <http://www.tera.org/Peer/VCCEP/Acetone/acevccep.pdf>.

Fowles, J., & Dybing, E. (2003). Application of toxicological risk assessment principles to the chemical constituents of cigarette smoke. *Tob Control, 12*(4), 424-430.

IRIS. (2017). Integrated Risk Information System. <https://www.epa.gov/iris>.

O'Brien, D. (1996). Michigan Department of Natural Resources, Initial Threshold Screening Levels (ITSL) for glycerin. <http://www.deq.state.mi.us/aps/downloads/ATSL/56-81-55/56-81-55_58hr_ITSL.pdf>.

Schaller, J.-P., Pijnenburg, J. P. M., Ajithkumar, A., & Tricker, A. R. (2016). Evaluation of the Tobacco Heating System 2.2. Part 3: Influence of the tobacco blend on the formation of harmful and potentially harmful constituents of the Tobacco Heating System 2.2 aerosol. *Regulatory Toxicology and Pharmacology, 81, Supplement 2*, S48-S58. doi:<http://dx.doi.org/10.1016/j.yrtph.2016.10.016>

Stephens, W. E. (2017). Comparing the cancer potencies of emissions from vapourised nicotine products including e-cigarettes with those of tobacco smoke. *Tob Control*. doi:10.1136/tobaccocontrol-2017-053808

Talhout, R., Schulz, T., Florek, E., van Benthem, J., Wester, P., & Opperhuizen, A. (2011). Hazardous compounds in tobacco smoke. *Int J Environ Res Public Health, 8*(2), 613-628. doi:10.3390/ijerph8020613

TCEQ. (2015). Texas Commission on Environmental Quality (TCEQ), Crotonaldehyde (Cis and Trans) Development Support Document. <https://www.tceq.texas.gov/assets/public/implementation/tox/dsd/final/sept15/crotonaldehyde.pdf>.

WHO. (1994). World Health Organization. Assessing Human Health Risk of Chemicals: Derivation of Guidance Values for Health-Based Exposure Limits. World Health Organization, Geneva. *Environmental Health Criteria. , 170*.

WHO. (2000a). World Health Organization (WHO): Air Quality Guidelines for Europe. <http://www.euro.who.int/__data/assets/pdf_file/0005/74732/E71922.pdf>.

WHO. (2000b). World Health Organization (WHO): Air Quality Guidelines for Europe Second Edition. <http://www.euro.who.int/__data/assets/pdf_file/0005/74732/E71922.pdf>.
