# Supplementary material for: A Method for Comparing the Impact on Carcinogenicity of Tobacco Products: A Case Study on Heated Tobacco Versus Cigarettes
Source: Risk Anal. 2020 May 1;40(7):1355–66. doi: 10.1111/risa.13482 (PMC7496151; doi:10.1111/risa.13482)
Supplement: Supplementary file 2 — Supplementary Material [file RISA-40-1355-s002.docx]

**Supplemental Material 1 (SM1) to “A method for comparing the impact on carcinogenicity of tobacco products: a case study on heated tobacco versus cigarettes” by W. Slob et al.**

**Dose-response analyses**

To illustrate the BMD analyses in more detail, some of the intermediate results are shown below.

The analyses were performed for each of the four severity categories 2 - 5 (see main text) separately.

All datasets for a given severity category were treated as one combined dataset, while each individual dataset (i.e. with all levels of the concomitant factors the same) was used as a covariate in the dose-response analysis. In particular, the scaling parameters of the dose-response models (*a* = response in controls, and *b* = potency) were estimated for each dataset separately, while the shape parameters were assumed to be constant among datasets. This implies that the fitted curves to each dataset (in the same combined dataset) have the same shape, i.e. they are parallel on log-dose scale. Since the graphs below do not indicate that the fitted curves deviate from the data, the assumption of parallel curves is not rejected.

Datasets associated with a study duration of less than 96 day were excluded. Furthermore, in butadiene doses 362 and 378 mg/kg (males) and dose 435 mg/kg (females) resulted in substantial decrease in response (after an initial increase), and these doses were omitted as well.

**PART I. Determination of critical dataset for each compound (and severity category)**

For each of the severity scores 2 to 5, the log-logistic model was fitted to all the individual datasets combined, using dataset as a covariate in fitting the model (software: PROAST version 65.12). The expression of the log-logistic model is

*y* = *a* + (1 - *a*) / { 1 + exp[*c* ln(*b*/*x*)] } (1)

where *y* is the response (incidence), *x* the dose, and *a*, *b*, and *c* are constants to be estimated by fitting the model to the data. We assumed that parameters *a* (incidence at dose zero) and *b* (potency) depend on the covariate (individual dataset, as defined above), while parameter *c* is the same among datasets. Parameter *c* reflects the steepness of the dose-response, and assuming that its value is the same among similar datasets has been confirmed in various similar analyses (Braakhuis et al., 2018; Slob & Setzer, 2014; Soeteman-Hernandez, Johnson, & Slob, 2016).

The following graphs show, for each severity category, the fit of the overall (log-logistic) model to the combined dataset, followed by a number of plots showing the same results, but plotted for each compound separately (to improve readability). The confidence intervals for the BMD were calculated for each individual dataset, based on these combined model fits.

SEVERITY 2: Benign tumor(s)


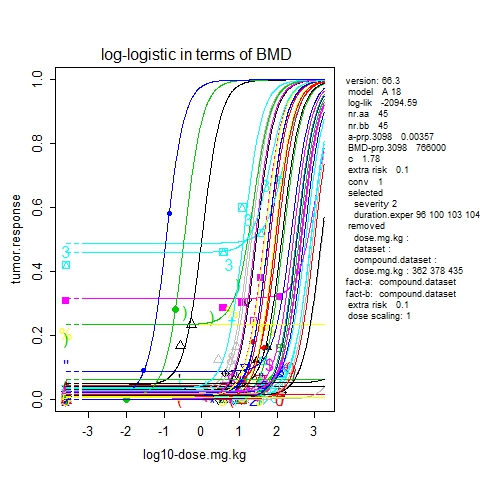

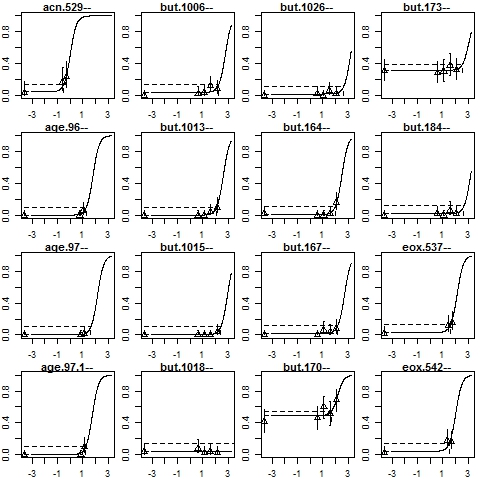

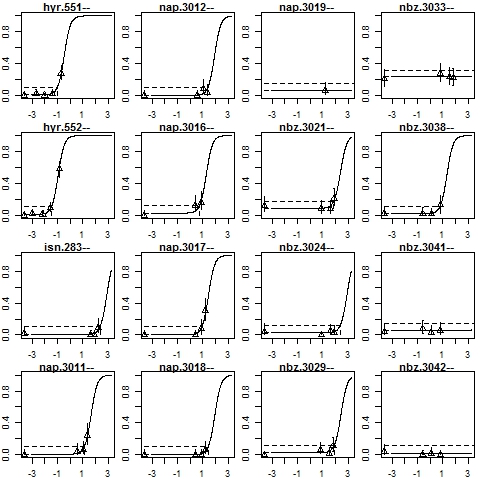

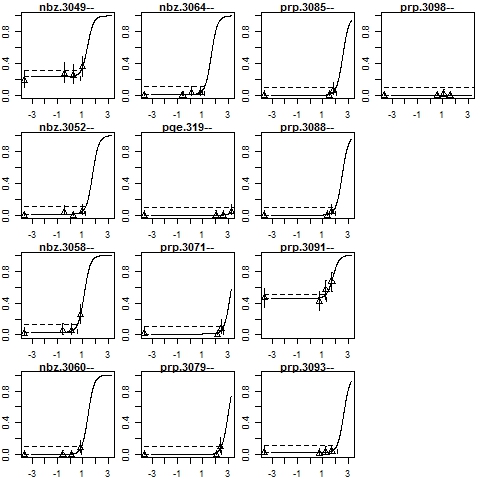


SEVERITY 3: Malignant tumor(s) in a single organ


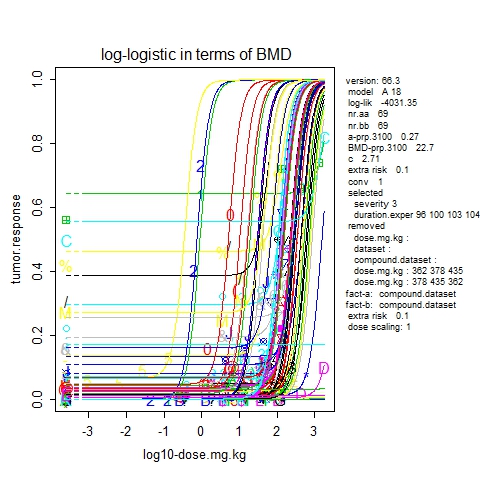

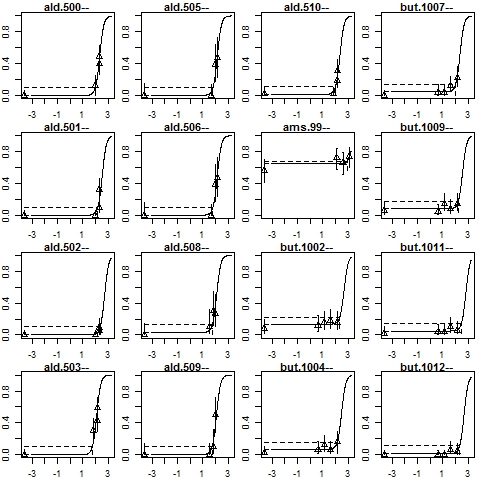


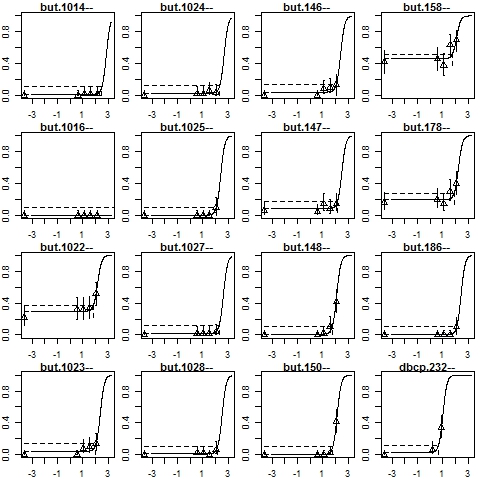

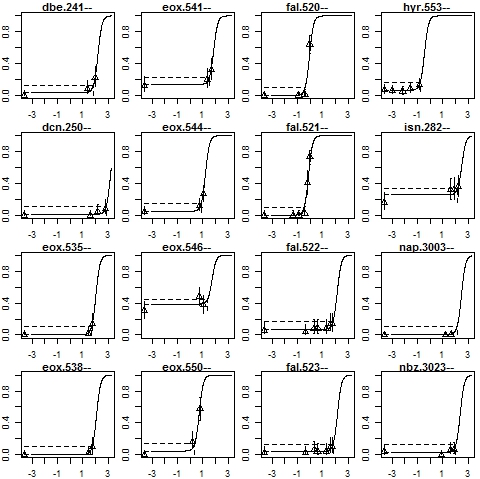

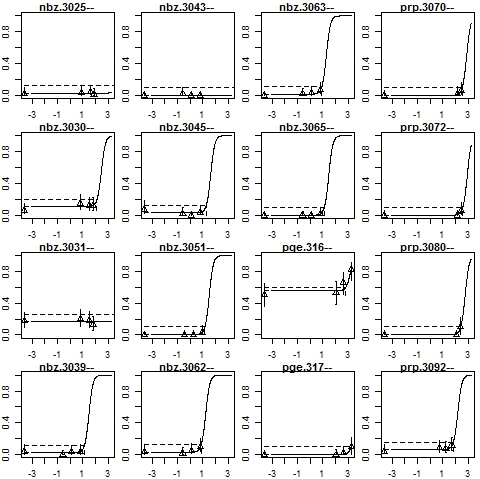

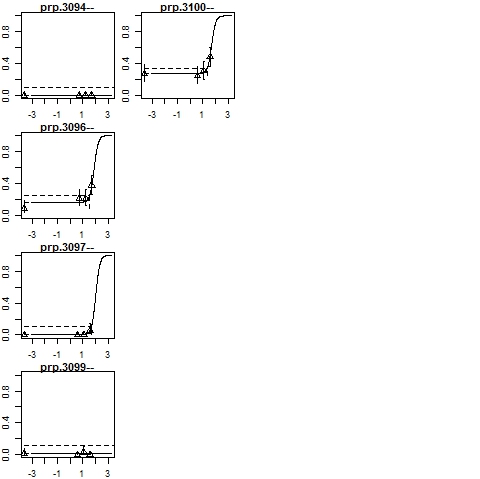


SEVERITY 4: Malignant (metastasizing) tumors in different organs

SEVERITY 5: TBA

Based on the dose-response analyses above, the BMD confidence intervals were calculated for each individual dataset. These are plotted below, again for each severity category. The dashed intervals in these plots have at least one infinite bound (note that 0 is an infinite lower bound).

For each severity category, the “critical” dataset is selected for each compound, based on the lowest value for the geometric mean of the lower and upper bound of each BMD confidence interval available for that compound. BMD CIs with infinite bounds (see the dashed intervals in the figures above) were not included. These “critical” datasets were then combined for a second BMD analysis, as shown in PART II.

**PART II. Estimation of RPFs**

The combined critical datasets for each combined were used to derive the RPFs, by fitting the exponential latent variable model (LVM)

*y* = *a* [*c* - (*c* - 1)exp(- *b*x*^d^*)] (2)

where *y* is the latent variable, and parameters *a*, *b*, *c*, and *d* are constants estimated by fitting the model. Parameters *a* and *b* are assumed to depend on the covariate (compound), while parameter *d* (reflecting the steepness of the dose-response) is assumed constant among dataset. Parameter *c* is assumed constant as well, but results in a low value, which translates into a maximum incidence of around 100% for all datasets. In other words, the assumption is that the dose-response would always approach 100% of the animals responding, if dose were high enough.

More specifically, we applied this model in its re-parameterized version such that it contains the BMD for the reference compound, and RPFs for each of the other compounds (see model 32 in PROAST). The next figures show, for each category, the fitted model to the selected datasets per compound.

SEVERITY 2:

SEVERITY 3

SEVERITY 4:

SEVERITY 5

These analyses resulted in the following confidence intervals for the RPFs (and for the CED for butadiene).

The confidence intervals for the RPFs (and BMD for butadiene) are summarized in the table below. These values were used to derive the final uncertainty bounds for the RPFs, according to the following procedure.

Virtually all RPF confidence intervals established for the same compound but related to different severity categories overlapped. The four confidence intervals of the BMD for the reference compound (1,3-butadiene) similarly overlapped except that the confidence interval for category 5 did not overlap with that for category 3. Category 5 is not a well-defined category from a biological point of view, as tumor-bearing animals can relate to various carcinogenic endpoints, with differing degrees of severity. Thus, we took category 3, being a relatively well-defined category containing most of the compounds, as the basis for deriving the overall compound-specific RPFs. However, if the upper bound in another category was higher, we used that value for the upper bound (i.e. this was considered additional uncertainty). For those compounds that were missing in category 3 we took the lowest lower bound and highest upper bound from the other categories (as far as available). This resulted in the confidence intervals given in Table 2 of the main text.

| sev cat 2 |  |  |  | sev cat 3 |  |  |  | sev cat 4 |  |  |  | sev cat 5 |  |  |
| --- | --- | --- | --- | --- | --- | --- | --- | --- | --- | --- | --- | --- | --- | --- |
|  |  |  |  |  |  |  |  |  |  |  |  |  |  |  |
| RPF-acn.529 | 5.76 | 182 |  |  |  |  |  |  |  |  |  | RPF-acn.530 | 26.2 | 192 |
| RPF-age.97.1 | 0.089 | 2.69 |  |  |  |  |  |  |  |  |  | RPF-age.96.1 | 0 | 1.86 |
|  |  |  |  | RPF-ald.509 | 0.469 | 0.931 |  |  |  |  |  | RPF-ald.507 | 0.0699 | 0.614 |
|  |  |  |  | RPF-ams.99 | 0.583 | 5.39 |  |  |  |  |  | RPF-ams.98 | 0.0327 | 0.236 |
|  |  |  |  |  |  |  |  | RPF-bap.3103 | 29.3 | 187 |  |  |  |  |
| BMD-but.170 | 1.33 | 38.4 |  | BMD-but.148 | 30.3 | 56 |  | BMD-but.181 | 12.5 | 36.3 |  | BMD-but.1029 | 2.63 | 19.3 |
|  |  |  |  | RPF-dbcp.232 | 8.95 | 25.6 |  | RPF-dbcp.230 | 80.2 | 838 |  |  |  |  |
|  |  |  |  | RPF-dbe.241 | 0.575 | 1.67 |  | RPF-dbe.237 | 15 | 286 |  | RPF-dbe.239 | 0.139 | 1.05 |
|  |  |  |  | RPF-dcn.250 | 0.0175 | 0.11 |  |  |  |  |  | RPF-dcn.249 | 0 | 0.0857 |
| RPF-eox.542 | 0.0548 | 1.61 |  | RPF-eox.550 | 21.7 | 71.5 |  |  |  |  |  | RPF-eox.549 | 2.97 | 21.3 |
|  |  |  |  | RPF-fal.521 | 68.2 | 129 |  |  |  |  |  | RPF-fal.524 | 0 | 0.173 |
| RPF-hyr.552 | 47.1 | 1120 |  | RPF-hyr.553 | 5.65 | 560 |  |  |  |  |  |  |  |  |
| RPF-isn.283 | 0.000649 | 0.17 |  | RPF-isn.282 | 0.176 | 3.7 |  |  |  |  |  | RPF-isn.284 | 0.0397 | 0.439 |
| RPF-nap.3016 | 0.339 | 8.02 |  | RPF-nap.3003 | 0 | 0.726 |  |  |  |  |  | RPF-nap.3015 | 0.69 | 5.05 |
| RPF-nbz.3058 | 0.551 | 13.4 |  | RPF-nbz.3062 | 0.115 | 11.3 |  |  |  |  |  | RPF-nbz.3057 | 1.05 | 7.36 |
| RPF-pge.319 | 0.000437 | 0.0183 |  | RPF-pge.316 | 0.61 | 5.9 |  |  |  |  |  | RPF-pge.318 | 0.0449 | 0.237 |
| RPF-prp.3091 | 0.362 | 11.7 |  | RPF-prp.3100 | 1.85 | 22.8 |  |  |  |  |  | RPF-prp.3095 | 7.15 | 41 |

Braakhuis, H. M., Slob, W., Olthof, E. D., Wolterink, G., Zwart, E. P., Gremmer, E. R., . . . Luijten, M. (2018). Is current risk assessment of non-genotoxic carcinogens protective? *Crit Rev Toxicol, 48*(6), 500-511. doi:10.1080/10408444.2018.1458818

Slob, W., & Setzer, R. W. (2014). Shape and steepness of toxicological dose-response relationships of continuous endpoints. *Crit Rev Toxicol, 44*(3), 270-297. Retrieved from <http://informahealthcare.com/doi/abs/10.3109/10408444.2013.853726>

Soeteman-Hernandez, L. G., Johnson, G. E., & Slob, W. (2016). Estimating the carcinogenic potency of chemicals from the in vivo micronucleus test. *Mutagenesis, 31*(3), 347-358. doi:10.1093/mutage/gev043
