# Supplementary material for: A Method for Comparing the Impact on Carcinogenicity of Tobacco Products: A Case Study on Heated Tobacco Versus Cigarettes
Source: Risk Anal. 2020 May 1;40(7):1355–66. doi: 10.1111/risa.13482 (PMC7496151; doi:10.1111/risa.13482)
Supplement: Supplementary file 3 — Supplementary Material [file RISA-40-1355-s003.docx]

**Supplemental Material 2 (SM2) to “A method for comparing the impact on carcinogenicity of tobacco products: a case study on heated tobacco versus cigarettes” by W. Slob et al.**

Calculation of the lower and upper uncertainty bounds (last two columns) for the (geometric) mean emissions for 9 compounds measured in the tobacco heating system (THS; see first four columns). The factor k (9th column) is the width of the lognormal uncertainty distribution expressed as the 95th percentile to the GM (or the GM to the 5th percentile).

|  | Chemical name | THS FR (R) 2.2 ug/stick | THS D2 (R) 2.2 ug/stick | THS FR (M) 2.2 ug/stick | THS D1 (M) 2.2 ug/stick | mean of  ln-emissions | var of  ln-emissions | k of CI | LB of geometric mean emission | UB of geometric mean emission |
| --- | --- | --- | --- | --- | --- | --- | --- | --- | --- | --- |
| acn | Acrylonitrile | 0.258 | 0.186 | 0.220 | 0.196 | -1.55 | 0.0210 | 1.186 | 1.80E-01 | 2.53E-01 |
| ald | Acetaldehyde | 219 | 213 | 205 | 220 | 5.37 | 0.00105 | 1.039 | 2.06E+02 | 2.22E+02 |
| but | 1,3-Butadiene | 0.294 | 0.319 | 0.265 | 0.411 | -1.15 | 0.03507 | 1.246 | 2.55E-01 | 3.96E-01 |
| eox | Ethylene oxide | 0.201 | 0.314 | 0.202 | 0.273 | -1.42 | 0.0498 | 1.300 | 1.87E-01 | 3.16E-01 |
| fal | Formaldehyde | 5.530 | 5.220 | 4.550 | 6.190 | 1.68 | 0.01639 | 1.162 | 4.59E+00 | 6.21E+00 |
| bap | Benzo[a]pyrene | 0.0001* | 0.00190 | 0.00129 | 0.00108 | -7.24 | 1.781 | 4.797 | 1.50E-04 | 3.44E-03 |
| nbz | Nitrobenzene | 1.88E-04 * | 9.2E-05 | 3.35E-04 | 1.55E-04 | -8.66 | 0.2848 | 1.872 | 9.25E-05 | 3.24E-04 |
| prp | Propylene oxide | 0.148 | 0.175 | 0.149 | 0.14 | -1.88 | 0.00923 | 1.120 | 1.36E-01 | 1.71E-01 |

- Measurement was below LOQ, and value represents the LOQ.

Calculation of the lower and upper uncertainty bounds (last two columns) for the (geometric) mean emissions for 9 compounds measured in a reference cigarette (see first two columns). The factor k (6th column) is the width of the lognormal uncertainty distribution expressed as the 95th percentile to the GM (or the GM to the 5th percentile).

|  | 3R4F ug/cigarette | 3R4F ug/cigarette | mean of  ln-emissions | var of  ln-emissions | k of CI | LB of geometric mean emission | UB of geometric mean emission |
| --- | --- | --- | --- | --- | --- | --- | --- |
| acn | 31.90 | 31.60 | 3.46 | 4.464E-05 | 1.030 | 30.82 | 32.71 |
| ald | 1555.00 | 1589.00 | 7.36 | 2.339E-04 | 1.071 | 1468.38 | 1682.74 |
| but | 63.80 | 91.80 | 4.34 | 6.620E-02 | 3.146 | 24.33 | 240.77 |
| eox | 29.40 | 34.20 | 3.46 | 1.144E-02 | 1.610 | 19.69 | 51.06 |
| fal | 56.50 | 68.70 | 4.13 | 1.911E-02 | 1.851 | 33.65 | 115.34 |
| bap | 0.0142 | 0.0137 | -4.27 | 6.425E-04 | 1.120 | 0.01 | 0.02 |
| nbz | 0.0086 | 0.00055 | -6.13 | 3.787E+00 | 5817.085 | 0.00 | 12.67 |
| prp | 1.32 | 1.72 | 0.41 | 3.503E-02 | 2.302 | 0.65 | 3.47 |
